# Older Persons’ and Health Care Professionals’ Design Choices When Co-Designing a Medication Plan Aiming to Promote Patient Safety: Case Study

**DOI:** 10.2196/49154

**Published:** 2023-10-05

**Authors:** Malin Holmqvist, Linda Johansson, Bertil Lindenfalk, Johan Thor, Axel Ros

**Affiliations:** 1 Department of Public Health and Healthcare Region Jönköping County Jönköping Sweden; 2 School of Health and Welfare Jönköping University Jönköping Sweden; 3 Institute of Gerontology School of Health and Welfare Jönköping University Jönköping Sweden; 4 Jönköping Academy for Improvement of Health and Welfare School of Health and Welfare Jönköping University Jönköping Sweden; 5 Futurum Region Jönköping County Jönköping Sweden

**Keywords:** co-design, engagement, medications, medication plan, older people, older adults, participatory, patient experience, patient safety, remote

## Abstract

**Background:**

Harm from medications is a major patient safety challenge among older persons. Adverse drug events tend to arise when prescribing or evaluating medications; therefore, interventions targeting these may promote patient safety. Guidelines highlight the value of a joint plan for continued treatment. If such a plan includes medications, a medication plan promoting patient safety is advised. There is growing evidence for the benefits of including patients and health care professionals in initiatives for improving health care products and services through co-design.

**Objective:**

This study aimed to identify participants’ needs and requirements for a medication plan and explore their reasoning for different design choices.

**Methods:**

Using a case study design, we collected and analyzed qualitative and quantitative data and compared them side by side. We explored the needs and requirements for a medication plan expressed by 14 participants (older persons, nurses, and physicians) during a co-design initiative in a regional health system in Sweden. We performed a directed content analysis of qualitative data gathered from co-design sessions and interviews. Descriptive statistics were used to analyze the quantitative data from survey answers.

**Results:**

A medication plan must provide an added everyday value related to safety, effort, and engagement. The physicians addressed challenges in setting aside time to apply a medication plan, whereas the older persons raised the potential for increased patient involvement. According to the participants, a medication plan needs to support communication, continuity, and interaction. The nurses specifically addressed the need for a plan that was easy to gain an overview of. Important function requirements included providing instant access, automation, and attention. Content requirements included providing detailed information about the medication treatment. Having the plan linked to the medication list and instantly obtainable information was also requested.

**Conclusions:**

After discussing the needs and requirements for a medication plan, the participants agreed on an iteratively developed medication plan prototype linked to the medication list within the existing electronic health record. According to the participants, the medication plan prototype may promote patient safety and enable patient engagement, but concerns were raised about its use in daily clinical practice. The last step in the co-design framework is testing the intervention to explore how it works and connects with users. Therefore, testing the medication plan prototype in clinical practice would be a future step.

## Introduction

### Supporting Patient Safety

Patient safety, referred to as the prevention of harm to patients [1], is essential in health care. Older people are at an increased risk for adverse drug events (ADEs), harm caused by the use of medications [2,3], as they have a higher prevalence of frailty, multiple medical conditions, and polypharmacy [4,5]. Polypharmacy is commonly referred to as the use of multiple medications, but there is no universally accepted definition. An alternative definition is the use of more medications than medically necessary [6,7]. ADEs tend to occur during the entirety of medication use, but for older people in ambulatory care, most ADEs tend to arise when their medications are prescribed or evaluated [8]. Therefore, interventions targeting these steps may promote patient safety.

In an interview study with older people about how they experienced the evaluation of their medications, we found that they wanted to be involved in their care, and they called for specific written information regarding plans for the evaluation of their medications [9]. Patients participating in their own care may help prevent adverse events and can be seen as a source of insight, enhancing the safety of health care [10]. Patients and their next of kin can detect changes in patients’ condition, and if health care professionals enable them to interact, these signals may help optimize medication treatment [11]. Nurses and physicians identify good communication among persons involved in an older person’s medication treatment as a facilitator of proper evaluation [12]. To support safe treatment, pharmaceutical information, such as medication lists and care plans, can be shared among health care professionals [13,14].

International guidelines targeting multimorbidity and polypharmacy in older people highlight the value of a joint plan for continued treatment, that is, a “medication plan” for both older people and health care professionals to facilitate safer medication treatment [15]. Moreover, an agreement between older persons who use medications and health care professionals on health-related goals for treatment may benefit all those involved and prevent harm [16-18]. In a similar spirit, Sweden has a national program for the implementation of “Patient Contracts” [19,20], an agreement regarding the patient’s planned health care, created collaboratively, documented in the electronic health record (EHR), and intended to strengthen the relationship between a patient and health care professionals. To work effectively in clinical practice, a joint plan must meet the needs of potential users. So far, the needs and requirements for a medication plan, as expressed by patients and health care professionals, have not informed such plans.

### Co-Designing a Medication Plan

There is growing evidence for the benefits of including patients and health care professionals in initiatives for improving health care products and services [21]. Specifically, co-design is a way to improve health care that offers health care organizations new ways of creating services or products by harnessing the experiences of patients and health professionals [22]. Co-design has been integrated into improvement projects to develop interventions that enhance medication safety and has been recognized as a useful approach that puts the users’ input at the center [23]. There are several models for co-design, all focusing on the lived experiences of the participants and encouraging collaborative work to identify problems and solutions [24,25]. Therefore, we first explored older persons’ and health care professionals’ experiences of the evaluation of medications [9,12]. On the basis of these findings, a remote co-design initiative involving older persons, physicians, and nurses was applied to define and develop a medication plan with the aim of supporting medication evaluation. In a previous study, we found that the participants experienced the remotely completed co-design initiative to be inclusive, to facilitate learning, and to increase opportunities to collaboratively design a medication plan [26]. This study aimed to identify the participants’ needs and requirements for a medication plan and explore their reasoning for different design choices.

## Methods

### Study Design

A case study design was used, as it is useful when studying improvement efforts in complex systems such as health care [27,28]. According to the case study approach, qualitative and quantitative data were first analyzed separately and then compared side by side in the *Discussion* section [27].

### Setting and Participants

The co-design initiative was established in 1 of the 21 regional public health care systems in Sweden. Most health care organizations in Sweden use EHRs. Access to medical data is regulated by the Patient Data Law [29]. Each health care organization has its own EHR but can share data, for example, medical notes or lists of prescribed medications, with the National Patient Overview (NPO) [30], which gives authorized health care professionals access to medical information about a patient previously cared for elsewhere. In addition, patients can access their own EHR digitally through the secure web interface 1177 [30]. In Sweden, electronic prescriptions are standard and visible to patients and authorized health care professionals through the Swedish National Medication List [31]. For patients with multiple dose drug dispensing support, prescriptions are managed in a web-based service available to authorized health care professionals [30].

Participants were recruited through existing contacts within the regional public health care system’s office for the Patient Contracts program. To reach a variety of perspectives and experiences [32], we sought a group including older persons (aged >75 years) with lived experience of taking long-term medications, next of kin, general practitioners, and nurses working in municipality-based home health care. Inclusion required adequate communication capability in Swedish, access to and proficiency in using the internet, and the possibility to participate in all 3 parts of the co-design initiative. No exclusion criteria were applied. For the older persons, we noted gender, age, and the number of current medications; for the health care professionals, we noted gender and years in the profession. The initiative involved 14 participants, namely 5 (36%) older persons aged 72 to 82 years using 3 to 8 medications daily, 6 (43%) nurses who had worked for 4 to 35 years, and 3 (21%) physicians who had worked for 5 to 39 years. We did not succeed in including next of kin through the existing contacts, but one of the older persons reported also having the experience of being next of kin.

### The Co-Design Initiative

**Figure 1:**
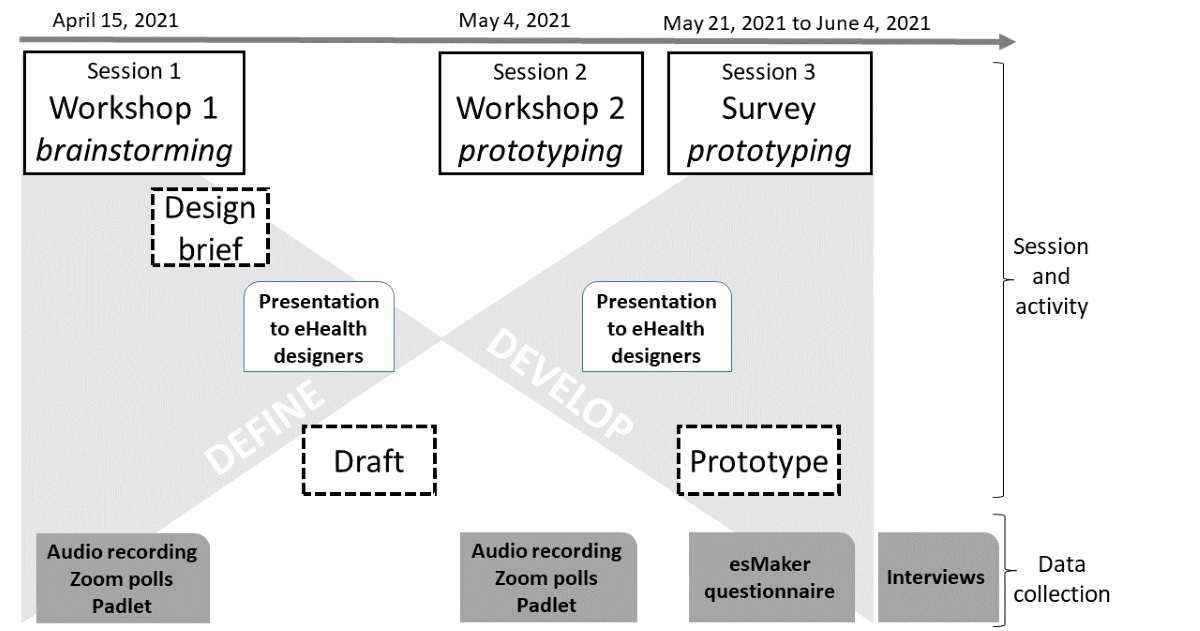
The structure of the co-design initiative and data collection.

The “Double Diamond” framework, which consists of 4 phases, namely *Discover*, *Define*, *Develop*, and *Deliver* [33], was used to co-design a medication plan prototype, that is, a model of a proposed solution, incorporated within the existing EHR structure. The co-design initiative, involving the define and develop phases in the double diamond framework, is described in detail elsewhere [26]. As the COVID-19 pandemic brought restrictions on physical meetings, the initiative was performed remotely digitally over a 2-month period (Figure 1). It included 3 sessions: 2 time-scheduled workshops lasting 2 hours each conducted via web-supported Zoom videoconferencing software (Zoom Video Communications, Inc) and 1 web-based survey. A quality improvement adviser and the first author facilitated the workshops. eHealth designers in the regional public health care system prepared drafts and the prototype between the sessions based on outputs.

In the *Define* phase including the first session, workshop 1, insights from older persons, nurses, and physicians identified in the *Discover* phase [9,12] were presented to the participants along with information from research and regulations related to the initiative topic. The participants were asked to describe their needs for the medication plan, that is, what the medication plan must satisfy for them to get the right outcome [34] or, in practice, what the medication plan should contribute and add to existing practice. Then, they were asked to identify function and content requirements for the medication plan. Function requirements were the specific functionalities wanted for the medication plan to support its usability, and content requirements were the various pieces of information wanted in the medication plan [34]. Brainstorming was used to gather ideas and build a shared understanding of the orientation of the group. After workshop 1, the first author prepared a written design brief, which was a core reference point, based on the data gathered during workshop 1. The design brief was presented to the eHealth designers, who used it to prepare medication plan drafts, which were preliminary prototypes presented as a Microsoft Word (Microsoft Corp) document with 3 different images for each draft: 1 image from the EHR in the regional health care system, 1 image from NPO and 1177, and 1 image as a paper-printed copy.

The *Develop* phase included sessions 2 and 3, workshop 2 and a survey. In workshop 2, the drafts were presented to the participants, who were invited to develop the drafts further into 1 prototype by designing the components in detail and iteratively refining the drafts. Experience prototyping [35], a way to test and refine a solution in interactive feedback loops, using fictitious patient cases, was used to enable the participants to gain first-hand understanding and receive feedback. After workshop 2, the first author gathered the data and presented them to the eHealth designers, further informing their design of the medication plan prototype. In the third session, the resulting prototype was sent to all the participants in a Word document together with the survey to collect final feedback on the prototype in a final feedback loop.

### Data Collection

This case study of the co-design initiative draws on both quantitative and qualitative data (Figure 1). The 2 workshops were audio recorded and then transcribed verbatim. During the workshops, the participants captured the discussions and their own reflections using notes on a digital notice board (Padlet web platform, Padlet). The notes were downloaded after each workshop. Zoom polls, that is, questions asked on Zoom, were used to narrow down the discussions and prioritize the needs, requirements, and final specifications. Overall, 9 Zoom polls were single-choice questions, and 7 were multiple-choice questions. To reflect the ongoing discussions, the prewritten Zoom polls were refined during each workshop by the first author. The design briefs, drafts, and prototype also constituted the case study data.

A survey, developed specifically for this study in the web-based survey tool esMaker NX3 (Entergate), was sent to all participants in the third session to collect feedback and reflections on the prototype. It consisted of 2 yes or no questions with space to add free-text comments, 6 questions with response options on a 10-grade Likert scale and a “do not know” option, and 7 additional free-text questions (Multimedia Appendix 1). The participants were asked to respond within 2 weeks; they received reminders after 1 week and on the last day for completion.

After the initiative, all the participants were invited to participate in an individual semistructured interview on Zoom. The interview guide (Multimedia Appendix 2), developed by the research team based on the results of the survey, included questions about the prototype and the co-design process. A total of 7 participants, specifically 1 (14%) physician, 4 (57%) older persons, and 2 (29%) nurses, volunteered. The interviews were audio recorded, lasted between 21 and 46 (median 28.5) minutes, and were transcribed verbatim.

### Data Analysis

The qualitative data, that is, transcriptions from workshops and interviews, answers from free-text questions, and notes from Padlet, were gathered in NVivo software (QSR International) and analyzed through directed content analysis [36]. This method was chosen to broaden the understanding of the concepts used in relation to a medication plan. Following the method, the analysis started with the 3 predetermined and defined key concepts addressed in the co-design initiative, namely “needs,” “function requirements,” and “content requirements.” These 3 key concepts formed 1 main category each. The first author read the transcripts, notes, and free-text answers to the survey questions. Quotes representing the preformed main categories were highlighted and placed into the relevant main category. Similar quotes in each main category were put together in codes. Then, each code was reviewed and read through for a first impression. Quotes not relevant to that code were either uncoded or moved to another code. Codes with similar content were compared and grouped together by abstraction to generate subcategories. In addition, quotes relevant to the study aim but not to the 3 predetermined main categories were also highlighted. These quotes were analyzed by putting them together in codes and by abstraction, forming 3 subcategories in 1 additional main category. The results of the preliminary analysis were first presented to and discussed and refined with the last author. Then, the results were presented to and discussed and refined with the entire research group. Matrix coding queries [37] within NVivo were applied to the data, which assessed how the quotes from the older persons, nurses, and physicians underpinned the different design choices (the identified codes and categories) and how different design choices were expressed over time.

Quantitative data from Zoom polls and the survey were analyzed and summarized using descriptive statistics such as number, median, and range.

### Ethical Considerations

This study was approved by the Swedish Ethical Review Authority (dnr 2020-04781) and adheres to the Declaration of Helsinki [38]. All the participants received written information regarding the study and provided written consent before the first session. Data were deidentified to maintain confidentiality and were presented such that no single individual could be identified. Data from the study were kept secure in accordance with national and local routines.

## Results

### The Co-Designed Medication Plan Prototype

**Figure 2:**
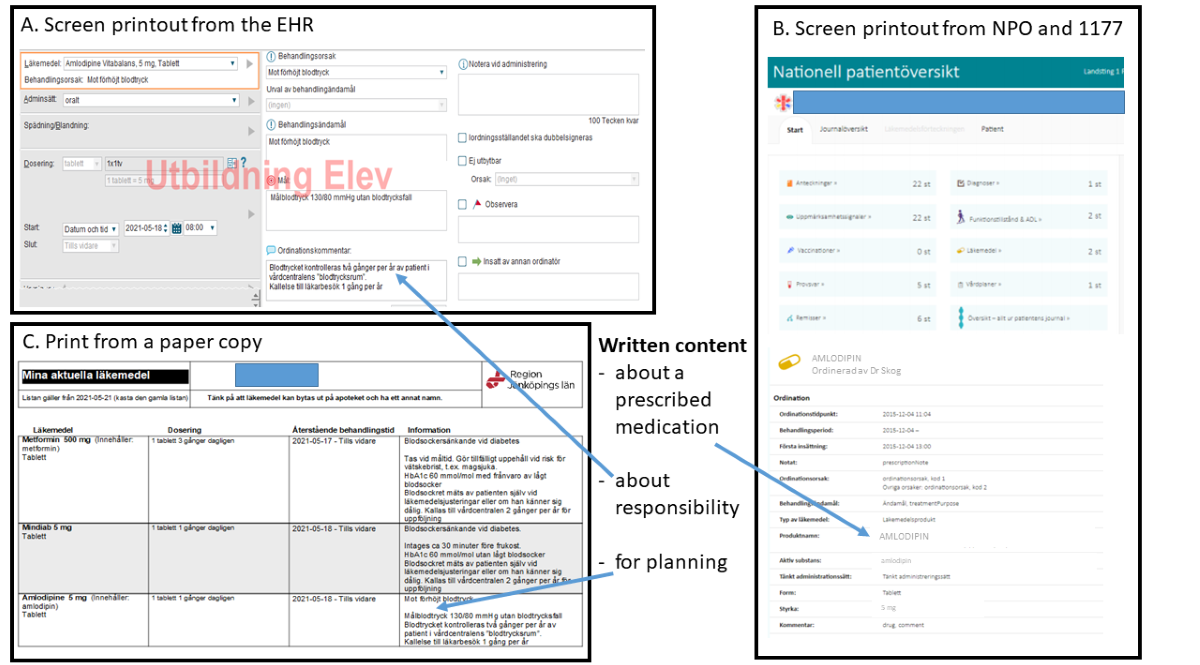
Illustration (in Swedish) of the content displayed in the final medication plan prototype: (A) electronic health record (EHR) screen printout; (B) National Patient Overview (NPO) and 1177 screen printout; and (C) print from a paper copy.

On the basis of the design brief with compiled information from Padlet notes and Zoom poll answers in the first session, the eHealth designers created 2 different drafts of a medication plan. One draft was based on the medication list, and the other draft took the form of a medical note. After refinements of the drafts, as suggested in workshop 2, the eHealth designers finalized a medication plan prototype derived from the medication list. The prototype was presented to the participants in the third session and remained intact after the survey (Figure 2).

### Qualitative Data Regarding the Participants’ Design Choices for the Medication Plan

#### Overview

The three predetermined key concepts formed the following three main categories: (1) *needs supporting communication, continuity, and interaction;* (2) *functions providing instant access, automation, and attention;* and (3) *content providing detailed information about the medication treatment*. Together with one additionally formed main category, (4) *the medication plan must provide added everyday value,* they described the participants’ reasoning for design choices for the medication plan. These main categories are presented in the subsequent sections with associated subcategories and codes as well as illustrative quotes.

#### Needs Supporting Communication, Continuity, and Interaction

During the initiative, the participants discussed the needs that the medication plan must meet to promote patient safety and work as intended. This means that the plan must support interaction and communication about the plan and be transparent and continuously updated. The needs are elaborated on in 3 subcategories: *adequate and adapted information, an updated and transparent source,* and *clarified responsibility and interaction* (Table 1).

A comparison of the data showed that the participants reasoned about the need for a medication plan mostly in the workshops, focusing more on the need for a balance of sufficient information and a plan that is easy to overview in workshop 2. Generally, the nurses had less input about needs, except for the need for a medication plan that is easy to gain an overview of. The physicians highlighted the need for a plan with a balanced amount of information. The older persons raised the need for understandable and clear information.

*Adequate and adapted information* that supports communication concerned striking a balance of sufficient information. It described the need to concentrate information into a reasonable amount and provide appropriately detailed information, as EHRs today tend to risk generating information overload. Exclusively, oral information is easy to forget; therefore, written information about the plan, printed on paper or digitally, is needed to allow reading afterward. This also allows other involved persons, who did not attend the visit, to take part in updates. In addition, the presentation of information must be adapted not only to the patient, but also to colleagues within health care to make it an understandable and clear plan.

*An updated and transparent source* reflected the need for the medication plan to be a living document, that is, to be continuously updated, for example, by updating it at the annual visit or when changing a patient’s medication. A medication plan can support the continuity of care if it is adapted to present conditions, which is important for older persons, in whom medical conditions can change quickly. There is also a need for the medication plan to be easy to gain an overview of, meaning that it should be clear and easy to find in a collected medical note. On the one hand, having the plan included in the medication list would make it easy to overview, but on the other hand, if many medications are prescribed, it may make the plan difficult to grasp.

*Clarified responsibility and interaction* addressed the need to make responsibilities visible, as who is doing what tends to be vague. The physician is primarily responsible for the prescribed medications, and patients trust the physician to take that responsibility. Even so, patients may have the responsibility to ensure that the treatment works as intended, provided that they know what to expect. In addition, home health care staff must know when they should support the older person. There is also a need for the medication plan to facilitate communication about treatment among the involved persons. Knowing whom to contact if there are questions or concerns promotes a sense of security. Mutual communication about medications may also help all those involved understand the next step in treatment.

**Table 1:** Overview of the main category *needs supporting communication, continuity, and interaction* with subcategories, codes, and illustrative quotes.

Subcategories and codes		Illustrative quotes
**Adequate and adapted information**		
	A balance of sufficient information	“There is already a lot of information in the medical record. It can be difficult to find the plan” (Group Padlet, workshop 2).
	An understandable and clear plan	“Sometimes it is written in a language that they do not understand, so it is also important that a non-medical language is used in the plan. If it says when they are going to evaluate the medicine, what it [the medication] is for in Swedish, when they are supposed to discontinue [the medication], then I think it will be easier” (Nurse, survey).
	Available written information	“I would like to have it in writing so I can remember it when I get home too” (Older person, workshop 1).
**An updated and transparent source**		
	Continuously updated	“It may be that when I start this plan, I may be quite alert. Then something happens and all of a sudden, I’m not that alert and then you might need to revise what applies again” (Nurse, workshop 1).
	Easy to gain an overview of	“We would like to have this collective contract...well, we have the same view as group one that it can be difficult to find in the medical record. There’s a lot in there from different...it’s...Some people have a lot of contacts and then it can be extremely difficult to find the medication list, if it’s only in the medical record” (Older person, workshop 2).
**Clarified responsibility and interaction**		
	Facilitate communication	“Enables better communication on why medicines are used and how they should be followed-up and by whom” (Group Padlet, workshop 2).
	Make responsibilities visible	“When prescribing, it is the doctor who is responsible for writing a plan and how to carry out the follow-up.” (Older persons, workshop 1).

#### Functions Providing Instant Access, Automation, and Attention

The participants specified the following function requirements for the medication plan to be usable: accessibility, embedded attention, and automation. The participants reasoned about these requirements, which are presented subsequently in 3 subcategories, namely *accessible for all involved, embedded alerts and communication,* and *automatically and instantly displayed* (Table 2).

A comparison of data showed that having the medication plan linked to the medication list was addressed mainly in workshop 2. Generally, the physicians addressed more function requirements. The older persons had fewer function requirements related to embedded alerts and automatically displayed information, something that the physicians addressed more. Instantly obtainable information was the major function requirement expressed by the nurses.

*Accessible for all involved* addressed a function of a connected EHR, meaning that EHRs from different health care providers have to be connected or at least should communicate with each other so that information is not lost in transition. Laws and regulations regarding confidentiality between caregivers may limit connections, and not all involved persons are digital today but probably will be in the future. According to the participants, this called for a printing option in the EHR to not exclude persons who do not have digital access at home or at work. However, there are challenges with printouts, as they may disappear, and it may be difficult to know which is the current one. The medication plan must also be readily accessible for eligible persons, such as authorized persons, including the older persons, next of kin, physicians, home health care staff, or even pharmacists. A function that makes the medication plan linked to the medication list could also make it more accessible, as this may facilitate management and the addition of complementary information to content that already exists there, thereby supporting an already existing structure.

**Table 2:** Overview of the main category *functions providing instant access, automation, and attention* with subcategories, codes, and illustrative quotes.

Subcategories and codes		Illustrative quotes
**Accessible for all involved**		
	A connected electronic health record.	“In the same medical record that everyone can access. And patients can also look on the web. Healthcare professionals must have the right information” (Older person, workshop 1).
	A printing option	“Digitally for healthcare professionals and in paper form for patients who need it” (Group Padlet, workshop 1).
	Readily accessible for eligible persons	“The patient in the first place, as we said, but also that relatives could access it if they are involved in the patient’s care. And, of course, healthcare professionals” (Nurse, workshop 1).
	Linked to the medication list	“It would be good if medication-related questions could be added to the medication list physically. If you hand it to the patient, that’s what you think. And also if it is sent or emailed to the municipality’s employees, it would be very good if you could comment directly on it” (Physician, workshop 1).
**Automatically and instantly displayed**		
	Automatic display of updated information	“It should be the same information throughout. No possibility of misunderstanding, and as you say here, four medical records! It is as if there is a risk of error” (Older person, workshop 1).
	Instantly obtainable information	“Risk if it is not easily obtainable to all healthcare professionals or if the information cannot be linked to Pascal [a web-based service for multiple dose drug dispensing]” (Group Padlet, workshop 2).
**Embedded alerts and communication**		
	A digital communication platform	“That you...as a user of medicines, can go in, and contact your doctor digitally, and say that it’s working well and...So you get this extra contact” (Older person, workshop 2).
	An embedded alert system	“This is somehow not all medications, but applies to some of them...Well, we need to be able to ‘flag’ which medications we should observe and which we do not need to be so observant about” (Physician, workshop 1).

*Automatically and instantly displayed* meant an automatic display of updated information, that is, not having to document the medication plan in several places in the medical record but having a function that copies text into places where it is needed. Ready-made suggestions for text phrases could simplify this, and it should be easy to see when the plan is updated. Having instantly obtainable information was requested, whereby the older persons, as well as health care professionals, can immediately read the medication plan. According to the participants, this is not always possible today, and easily obtaining information without having to go through unnecessary data creates a sense of security.

*Embedded alerts and communication* concerned having a digital communication platform where involved persons can communicate about the medication plan. Digital communication within the EHR already exists among health care professionals but should also enable older persons to communicate digitally in a secure manner. An embedded alert system that can draw attention to important issues in the plan and signal when a medication is altered or when it is time for follow-up was also raised as a desired function.

#### Content Providing Detailed Information About the Medication Treatment

The participants identified content requirements that could provide involved persons with detailed information about how to act and about the next step in treatment. These requirements were elaborated on in 3 subcategories: *written content about a prescribed medication, written content about responsibility,* and *written content for planning* (Table 3).

A comparison of the data showed that content requirements were mainly discussed during workshop 1, with a focus on planning. In workshop 2 and the survey, the content focused mainly on information about the prescribed medications. The older persons focused on content about the prescribed medications, the nurses focused on what to alert about, and the physicians focused on when to evaluate.

*Written content about a prescribed medication* described what the medication is used for and when to take the medication, that is, during the day or together with food and other medications. It also addressed the intended treatment duration and information about the refill of a prescription, that is, the quantity and number of withdrawals from the pharmacy.

*Written content about responsibility* described whom to contact for questions about the medication and who is responsible for follow-up and evaluation.

*Written content for planning* described how to monitor and evaluate, that is, the plan for evaluation, and treatment goals such as blood pressure targets. In addition, the participants called for information regarding what to alert about, such as potential side effects, and when to evaluate a medication, such as frequency or a date in a month.

**Table 3:** Overview of the main category *content providing detailed information about the medication plan* with subcategories, codes, and illustrative quotes.

Subcategories and codes		Illustrative quotes
**Written content about a prescribed medication**		
	Treatment duration	“And how long is the duration of the treatment” (Nurse, workshop 1).
	Refill of a prescription	“It would have been great if, in addition to the date, the quantity and number of withdrawals had been included in the list, as then the patient would have direct control over the prescriptions at the same time and could see that medicines are prescribed for a year” (Physician, survey).
	What the medication is used for	“And a short description of why to take the tablet” (Group Padlet, workshop 2).
	When to take the medication	“What many patients want to know is ‘How should they take their medicine?’ Should it be with a meal, with water, or when” (Older person, workshop 2).
**Written content about responsibility**		
	Who is responsible	“It is important that it is clear who. That is different responsibilities...What responsibility does the patient have and what responsibility we have as health care providers?” (Nurse, workshop 1)
	Whom to contact	“And it is not always necessary to have contact with a doctor, it can be a nurse” (Older person, workshop 2).
**Written content for planning**		
	How to monitor and evaluate	“And then you can discuss, either you have your own blood pressure monitor, or you go to our blood pressure room” (Physician, workshop 1).
	Treatment goal	“That it is important that the goal is clear, for example it is important that the minimum and maximum are stated for certain medicines, such as blood sugar levels” (Nurse, survey).
	What to alert about	“Potential side effects that may occur” (Nurse, workshop 1).
	When to evaluate	“And for my part, I think that it should be clearly written, when follow-up should take place” (Older person, workshop 1).

#### The Medication Plan Must Provide Added Everyday Value

According to the participants, a medication plan must provide added everyday value related to safety, effort, and engagement for older persons and health care professionals involved in daily clinical practice. The challenges and opportunities that the participants emphasized are elaborated upon in 3 subcategories: *challenges for clinical practice*, *enable patient engagement*, and *make medication treatment safer* (Table 4).

A comparison of the data showed that the physicians especially addressed challenges in prioritizing time wisely, if required to create medication plans, and challenges in individualizing the medication plan to each older person. Challenges in individualization were not addressed much by the older persons, who instead raised the possibility that a medication plan may empower patients to become more involved. During the initiative, enabling patient engagement was discussed more in workshop 1, whereas the challenges in applying the medication plan in clinical practice and safer medication treatment were discussed more in workshop 2.

*Challenges for clinical practice* reflected difficulties in applying a medication plan in a usable way in everyday clinical practice, where time can be scarce and the implementation of new ways of working can be difficult. Today, during a regular patient visit, physicians have limited time to prepare a medication plan. Introducing an additional task to the visit may generate stress and make it necessary to prioritize time wisely. Furthermore, using a medication plan may result in the older person having questions about their treatment, which may require additional time to handle. Therefore, introducing a medication plan may call for stepwise implementation, that is, for an implementation that is not rushed and tests the plan on a small scale, as innovations are not always welcomed in health care. To avoid shortcuts, such as not applying the medication plan properly, benefits such as enhanced safety must be highlighted.

*Enable patient engagement* addressed the opportunity that a medication plan provides to make older persons more involved in their medications; even so, there might be challenges, as older persons are a heterogeneous group, which calls for adaptions to their preferences and abilities. A medication plan can empower patient involvement if health care professionals invite the older person to engage in a dialog about their treatment. The older person can also take greater responsibility for their health and care, which might be desirable for both the older person and health care services. To enable patient engagement, it is necessary to individualize to suit the older person by making adjustments to every situation and every person involved, including the older person’s own capabilities and wishes as well as the physician’s preferences.

*Making medication treatment safer* dealt with the promotion of patient safety. The resources used for regular medication re-evaluation could be beneficial for safety. A shared understanding of the plan between the older persons and involved health care professionals can create security in collaboration and provide support for better medication re-evaluation. Although the medication plan may require resources, such as time to prepare and discuss the plan, it may reduce unnecessary care, such as unwanted admissions to hospitals or extra phone calls, if all involved persons know what to monitor and how to act in time before a complication related to medications evolves.

**Table 4:** Overview of the main category *the medication plan must provide added everyday value* with subcategories, codes, and illustrative quotes.

Subcategories and codes			Illustrative quotes
**Challenges for clinical practice**			
	Call for stepwise implementation	“Risk of not filling in everything [information] every time” (Group Padlet, workshop 2).	
	Prioritize time wisely	“There must be time to create the plan so the doctor does not rush it” (Nurse, survey).	
**Enable patient engagement**			
	Empower patient involvement	“I think it will activate us older people, so that we become more interested in our medicines” (Older person, workshop 2).	
	Individualize to suit the older person	“The older you are, the less you can take that responsibility. It may also be somewhat individual, how you put responsibility on the patient. At least I think so...But they have to know that [about their medications]” (Physician, workshop 1).	
**Make medication treatment safer**			
	Create security in collaboration	“I would feel more secure with information on how to take the medicine and when check-ups will take place and what measurements apply to each diagnosis” (Older person, survey).	
	Reduce unnecessary care	“I think you should still be able to spend some time on it because you probably gain a lot from it in the end. You avoid contact with us as well many times...To doctors when we ask things that could already have been answered” (Nurse, interview).	

### Quantitative Data Regarding the Participants’ Design Choices for the Medication Plan

*Zoom polls* conducted during workshop 1 were used to narrow down the participants’ views about what needs the medication plan must meet (Zoom polls 1-4) and their views about function and content requirements for the medication plan (Zoom polls 5-10). In workshop 1, 2 (14%) of the 14 participants participated together via the same computer, resulting in 13 respondents on the Zoom polls (Table 5). In workshop 2, Zoom polls were used to address the final specifications for the medication plan (Zoom polls 11-16). Moreover, in workshop 2, of 14 participants, the same 2 (14%) participants participated via the same computer, and 1 (7%) participant did not respond to the Zoom polls, resulting in 12 respondents (Table 5).

*The survey* was answered by 13 (93%) of the 14 participants. All (13/13, 100%) participants agreed that treatment goals and when and how treatment should be evaluated constituted the most important content in a medication plan and that this content was included in the prototype. In addition, everyone (13/13, 100%) agreed that the medication plan should be integrated into the medication list. In the questions with response options (Table 6), the older persons’ responses had higher median scores, and the nurses and physicians had lower median scores than those of the entire group. How well the prototype corresponded to a perfect medication plan had a slightly lower median score than the other questions.

**Table 5:** Presentation of the Zoom polls and the participants’ responses during the workshops.

Zoom polls				Responses, n (%)
**Needs for the medication plan (n=13)**				
	**1. What needs are most important for a medication plan to be safe and usable? (choice of 3)**			
		Easily available in the medical record	3 (23)	
		Information easy to understand	4 (31)	
		Same information to everyone involved	10 (77)	
		One way to communicate	2 (15)	
		Can be printed in paper	4 (31)	
		Clear agreement about responsibilities	11 (85)	
		“Contact person” for continuity	5 (38)	
	**2. How will a medication plan be accessible for those needing it? (multiple choices)**			
		Displayed in the EHR^a^ within the regional health care system	5 (38)	
		Be visible digitally at 1177^b^	10 (77)	
		Be visible digitally in the NPO^b,c^	8 (62)	
		Be printed on paper	4 (31)	
		Within the medication list	12 (92)	
		Do not know	0 (0)	
	**3. A clear division of responsibilities between persons included is (single choice)**			
		Very important	11 (85)	
		Important	2 (15)	
		Not that important	0 (0)	
		Unimportant	0 (0)	
		Do not know	0 (0)	
	**4. The medication plan must be completed during the visit to a physician (single choice)**			
		Yes, it should be ready to hand over at the visit.	6 (46)	
		No, it can be sent home or be available in 1177^b^ and NPO^b^ after the visit.	6 (46)	
		Unimportant	0 (0)	
		Do not know	1 (8)	
**Function and content requirements for the medication plan (n=13)**				
	**5. What information should be included in a medication plan? (multiple choices)**			
		Why the treatment is initiated	10 (77)	
		Treatment aim	13 (100)	
		When a medication will be reevaluated	11 (85)	
		How a medication will be followed up	9 (69)	
		Who will do the tests, measures, and take blood sample	8 (62)	
		Clear agreement	7 (54)	
		Who will follow up the treatment	10 (77)	
	**6. Who will use the medication plan? (multiple choices)**			
		The patient	13 (100)	
		Next of kin	12 (92)	
		Physicians	12 (92)	
		Nurses in home health care	12 (92)	
		Home care service staff	7 (54)	
		Care coordinators	13 (100)	
		Nurse in telephone counseling	8 (62)	
		Other	1 (8; pharmacist)	
	**7. When will the medication plan be used? (multiple choices)**			
		At the annual check-up at the primary care center	12 (92)	
		At all visits to the primary care center concerning medications	11 (85)	
		At home when you want to know the next step	7 (54)	
		In-home health care when planning care and treatment	12 (92)	
		Do not know	0 (0)	
	**8. How often should a medication plan be updated? (multiple choices)**			
		At each change of medication	12 (92)	
		At an annual check-up	9 (69)	
		At each physician’s visit	2 (15)	
		Unimportant	0 (0)	
		Do not know	0 (0)	
	**9. Where should the information be available? (single choice)**			
		Included in the medication list, visible in the health care provider’s EHR, at 1177^b^ and NPO^b^	5 (38)	
		As text in a medical note visible in the health care provider’s EHR, at 1177^b^ and NPO^b^	3 (23)	
		In a shared care plan, visible in the health care provider’s EHR, 1177^b^ and NPO^b^	2 (15)	
		Do not know	2 (15)	
	**10. Could a medication plan promote safer medication treatment? (single choice)**			
		Yes	8 (62)	
		Partly	5 (38)	
		No	0 (0)	
		Do not know	0 (0)	
**Specifications for the medication plan (n=12)**				
	**11. Do you agree with the summary presentation from the last session? (single choice)**			
		Fully agree	10 (83)	
		Agree	2 (17)	
		Partly agree	0 (0)	
		Do not agree	0 (0)	
	**12. Where should the medication plan be positioned in the EHR? (single choice)**			
		As a separate care plan within the medical notes	1 (8)	
		Within the medication list	11 (92)	
		Do not know	0 (0)	
	**13. What 3 keywords are the most important in a medication plan, to make it usable and safe? (multiple choices)**			
		Indication (why treatment is given)	6 (50)	
		Medication (and dosage)	12 (100)	
		Treatment aim	8 (67)	
		Effect and side effects	5 (42)	
		Duration of treatment	5 (42)	
		Planning and follow-up (in what way)	10 (83)	
		Responsibility	4 (33)	
	**14. If it takes 15 min to complete a medication plan, within a 45-min visit, which option do you prioritize? (single choice)**			
		The medication plan is documented during the visit and handed over directly	8 (67)	
		The medication plan is documented after the visit, available afterwards	2 (17)	
		Do not know	2 (17)	
	**15. How safe for patients does the medication plan feel? (single choice)**			
		0=not safe at all	0 (0)	
		1	1 (8)	
		2	10 (83)	
		3=very safe	0 (0)	
		Do not know	1 (8)	
	**16. How usable does the medication plan feel? (single choice)**			
		0=not usable at all	0 (0)	
		1	0 (0)	
		2	7 (58)	
		3=very usable	5 (42)	
		Do not know	0 (0)	

^a^EHR: electronic health record.

^b^Secure web interface where patients (1177) and health care professionals (National Patient Overview) can access EHR.

^c^NPO: National Patient Overview.

**Table 6:** Median scores from the survey with responses on a 10-grade Likert scale^a^.

Question	Total (n=13), median (IQR)	Older persons (n=5), median (IQR)	Nurses (n=5), median (IQR)	Physicians (n=3), median (IQR)
1. To what extent do you feel that the prototype meets your objectives for a medication plan?	9 (7-10)	10 (8-10)	8 (7-10)	9 (7-10)
2. Do you think that the time it would take to create or maintain a medication plan at a health care visit corresponds to its contribution to patient safety?^b^	9^b^ (7-10)	9.5^b^ (9-10)	9^b^ (8-10)	8 (7-9)
3. To what extent do you think that the medication plan may contribute to increased patient safety in medication treatment?	9 (7-10)	9 (7-10)	8 (7-10)	8 (7-9)
4. To what extent do you think the prototype is usable for you?	9 (6-10)	10 (9-10)	8 (6-10)	8 (2-9)
5. Would you consider using the prototype as a medication plan?	9 (7-10)	10 (9-10)	9 (7-10)	9 (7-10)
6. Imagine a perfect medication plan; how well does the prototype match your image?^c^	8^c^ (7-10)	9^c^ (8-10)	7 (7-9)	7 (7-8)

^a^Response on the Likert scale for questions 1 to 5: 1=do not agree and 10=totally agree; response on the Likert scale for question 6: 1=worst possible match and 10=best possible match.

^b^Three participants (n=2, 67% nurses and n=1, 33% older person chose “do not know”).

^c^One participant (1 older person did not answer).

## Discussion

### Principal Findings

In studying a co-design initiative, we explored how older persons, physicians, and nurses in home health care reasoned about different design choices that would make a medication plan work out in clinical practice and promote patient safety. The participants had partly diverging views about the needs and requirements for a medication plan; for instance, the older persons raised the need for understandable and clear information, and the physicians highlighted the need for a balanced amount of information. The nurses emphasized a function that could make information instantly obtainable. After reasoning about 2 generated drafts, they agreed on 1 medication plan prototype linked to the existing medication list (Figure 2). According to the participants, a medication plan needs to support communication, continuity, and interaction. To do so, they noted that information in the plan has to be adequate and adapted to all involved persons, which was further highlighted as important as the initiative progressed, as well as that the plan must be updated and transparent. An important function requirement that the participants agreed on and emphasized repeatedly was accessibility for all involved. The group defined accessibility as the possibility to share the plan easily within the EHR or as a printout. Embedded alerts and digital communication within the system, as well as automatically and instantly displayed information, were other key functions. Together with relevant medications, the participants found treatment aims and a plan for re-evaluation to be important content to include in the medication plan. Having a heterogeneous group of potential users reason together about the needs and requirements for the medication plan generated discussions on the potential everyday value of using the medication plan. The participants said that the medication plan had the potential to promote safer medication treatment and patient engagement, but they raised challenges related to its application and use in daily clinical practice.

### Comparison of Data and With Prior Work

Constantly updating the medication plan at annual visits at the primary care center or when medications were changed was prioritized by the participants according to the Zoom polls. Including the medication plan in the existing medication list was a requirement that was increasingly asked for over time according to both qualitative and quantitative data. Functions that made information instantly obtainable, automatically displayed, and updated were specified in general. The older persons asked for functions that would make the medication plan readily accessible to them and persons who support them. Sharing of and access to information similarly emerged as key issues in a qualitative study about patients’ perceptions of safety in primary care [39]. Likewise, an Australian co-design study addressing what older people want from integrated care showed that important aspects included the transfer of information among persons involved in a patient’s care [40]. Providing patients with access to their medical notes in the EHR improves their confidence in managing their own care [41], and digital health is increasingly embraced by older persons as well [42], indicating the importance of digital access to a medication plan for all those involved. In addition, the participating nurses raised the need for a medication plan that was easy to gain an overview of and with functions that made information instantly obtainable. The function requirement of having a plan obtainable via instant access to EHRs corresponded well with other studies performed with nurses in home health care in Sweden, as limited access to medication lists and medical record systems causes problems [43,44]. Variable access to medical records may also explain why the nurses in this study were not as satisfied as the other participants with how the final prototype met their expectations of a usable medication plan. In Sweden, comprehensive medical record keeping is regulated by a law on comprehensive health and care documentation [45], which allows organizations connected to the NPO to share medical information about a patient. The interoperability among EHRs seems to positively influence medication safety [46].

On the basis of all the participants’ responses to the Zoom polls, treatment aims, plans for follow-up, and clarity about responsibilities were prioritized content throughout the initiative. In addition, a need for the provision of consistent information in the medication plan to all those involved was emphasized. According to the qualitative data, the older persons prioritized content related to medications, whereas the health care professionals asked more specifically for content related to what situations to alert about and when to evaluate. Moreover, the older persons particularly highlighted the need for information that was understandable to them. The importance of receiving understandable information has also been reported in other studies addressing older persons’ experiences with information on medications [47] and their perceptions of safety [48,49]. Throughout the sessions, the physicians highlighted the need for a plan with a balance of sufficient information and with functions that could optimize documentation, for instance, by automatically copying information written in one note to other places in the EHR where the same information is needed. Since the implementation of EHRs in health care, there has been an ongoing debate addressing the physicians’ increasing workload related to excessive data entry requirements, long medical notes, and inaccessibility of information from other health care providers [50]. Moreover, questions about the amount of important therapeutic data in medical records have been addressed in an observational study in the Netherlands [51], exploring the ways in which therapeutic information in medical records is structured. Addressing the need for an optimal amount of data with a good structure is, therefore, important for a medication plan.

The participants highlighted the challenges and opportunities associated with a medication plan during the initiative. In the Zoom polls, the participants reflected on the notion that a medication plan would initially require extended time to create. In the discussions, the physicians especially addressed the challenges of not only prioritizing time but also individualizing the plan for each older person. The limited time during visits with older persons to discuss issues beyond acute problems and challenges in including their own goals and preferences into decisions around medications have been reported on previously [52] and will be important to address to make the medication plan work out well.

According to the survey, the older persons agreed more than the nurses and physicians that the medication plan might contribute to increased patient safety. The older persons also emphasized the potential for increased patient involvement. Empowering patient involvement and increased safety may be interrelated, as emerging evidence suggests that patients can, as cocreators of resilience, positively impact outcomes within health care [10]. To involve patients in both the health care they receive and the design of health care processes is in general both moral and logical according to O’Hara et al [53], as such involvement may support the resilience of the system. Having patients and others involved in the patient’s medication use process, knowing what to observe, when to act, who should act, and what actions they should take in case of deviation from the plan, can promote resilient performance [54,55], that is, the capacity to adapt to challenges and changes to maintain safety.

Even if the participants had partly diverging views during the initiative, their responses to the final survey showed that the medication plan prototype met their objectives to a large extent. In addition, they perceived that the prototype was jointly developed and accepted by consensus [26]. Undertaking a co-design process in which needs and requirements are specified can involve challenges [56,57] in achieving a shared understanding, managing the complexity of the different participants’ perspectives based on their different knowledge of the system, and transforming ideas into concrete functions. Even so, by involving users in the design of a medication plan, the chance that they will start using it in clinical practice increases [58].

### Strengths and Limitations

To ensure trustworthiness and that the findings in this case study mirrored the participants’ views of a medication plan prototype, we considered credibility, confirmability, dependability, and transferability throughout the analysis [59].

The co-design initiative involved 14 persons, namely older persons, physicians, and nurses in municipality-based home health care. The older persons we recruited, within the initiative Patient Contracts, may be considered as extra knowledgeable about and interested in strengthening patients’ role in health care. Persons who could not speak Swedish or who were unable to use a computer were excluded. Before starting, we hoped to involve next of kin as well, as they often play an important role in medication management for older persons [60]. The recruitment strategies and lack of the next-of-kin perspective can affect the *transferability* of the identified needs and requirements to other persons’ views. Testing the prototype will, therefore, be important to see whether it is consistent with other people’s views.

To support *dependability*, we conducted a pilot test of the setup for the initiative to determine whether the sessions allowed the participants to share their views about the needs and requirements for a medication plan. This resulted in some minor adjustments to the setup.

When considering the *confirmability* of data, objectivity is important. Therefore, peer debriefing was used in the directed content analysis, where the first and last authors refined the data and then presented the findings to and discussed the findings with the entire author group.

Finally, to ensure *credibility* and link the findings to reality, the drafts and findings from each session were shared continuously with the participants during the initiative. This allowed the participants to clarify their intentions, correct errors, and provide additional information in iterative loops. To ensure that the voices of all the participants were heard, the facilitators arranged moderated discussions. According to the participants, they were able to express their views during the co-design initiative and were listened to [26].

### Future Directions

The last step in the Double Diamond co-design framework [33], the *Delivery* phase, involves testing the co-designed intervention to explore how it works and connects with users in the setting it is intended for. Therefore, user testing of the medication plan prototype in clinical practice is a natural future step. This could be seen as a complex intervention, containing several interacting components and possibly producing varied outcomes, making it important to first test it on a small scale to find ways to collect data and evaluate outcomes [61].

### Conclusions

After reasoning about the needs and requirements for a medication plan, the participants agreed on an iteratively developed medication plan prototype linked to the medication list within the existing EHR. They stated that the needs for a medication plan are to support communication, continuity, and interaction; provide information that is adequate and adapted to everyone; and be easy to access and gain an overview of. According to the participants, the medication plan prototype may promote patient safety and enable patient engagement, but concerns were raised related to its use in daily clinical practice.

## Appendix

Multimedia Appendix 1 Survey.

Multimedia Appendix 2 Interview guide.

## Abbreviations

ADE: adverse drug event
EHR: electronic health record
NPO: National Patient Overview

## Data Availability

The data generated during this study are available from the corresponding author upon reasonable request.
